# Efficient single-pixel imaging based on a compact fiber laser array and untrained neural network

**DOI:** 10.1007/s12200-024-00112-8

**Published:** 2024-04-08

**Authors:** Wenchang Lai, Guozhong Lei, Qi Meng, Yan Wang, Yanxing Ma, Hao Liu, Wenda Cui, Kai Han

**Affiliations:** 1 College of Advanced Interdisciplinary Studies, National University of Defense Technology, Changsha, 410073 China; 2 Nanhu Laser Laboratory, National University of Defense Technology, Changsha, 410073 China

**Keywords:** Single-pixel imaging, Fiber laser array, Deep learning, Remote sensing

## Abstract

This paper presents an efficient scheme for single-pixel imaging (SPI) utilizing a phase-controlled fiber laser array and an untrained deep neural network. The fiber lasers are arranged in a compact hexagonal structure and coherently combined to generate illuminating light fields. Through the utilization of high-speed electro-optic modulators in each individual fiber laser module, the randomly modulated fiber laser array enables rapid speckle projection onto the object of interest. Furthermore, the untrained deep neural network is incorporated into the image reconstructing process to enhance the quality of the reconstructed images. Through simulations and experiments, we validate the feasibility of the proposed method and successfully achieve high-quality SPI utilizing the coherent fiber laser array at a sampling ratio of 1.6%. Given its potential for high emitting power and rapid modulation, the SPI scheme based on the fiber laser array holds promise for broad applications in remote sensing and other applicable fields.

**Graphical Abstract:**

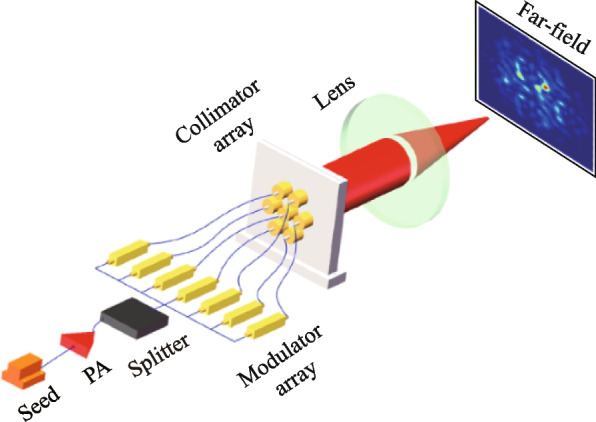

## Introduction

Single-pixel imaging (SPI) is an innovative computational imaging technique capable of reconstructing images from one-dimensional detector signals [1, 2]. Distinguished from traditional imaging methods employing array detectors like Charge Coupled Device (CCD), SPI requires structured illumination and a substantial number of measurements of the transmitted or reflected light intensities from the object of interest. By concurrently capturing the illuminating light fields and the reflected light intensities from the object, the reconstruction of the object can be achieved through computational algorithms. As most of the photons from the object are collected by the single-pixel detector, SPI exhibits high detection sensitivity and can operate across a wide spectral range. Consequently, SPI finds extensive applications in remote sensing [3, 4] non-visible imaging domains [5–9], optical computing [10, 11], optical encryption [12, 13], and so on.

SPI typically requires a significant number of single-pixel samples to reconstruct high-resolution images. To address this challenge and enable real-time imaging while reducing acquisition time, two main strategies are commonly employed. These strategies focus on improving the refresh rate of illuminating fields and developing efficient reconstruction algorithms. Several schemes have been reported to generate the illuminating light field, including the utilization of rotating ground glass [14, 15], digital micro-mirror device (DMD) [16, 17], liquid-crystal spatial light modulator (SLM) [18, 19], LED array [20, 21], and silicon-based optical phased array (OPA) chip [22, 23]. Notably, DMDs are capable of achieving refresh rates of up to 22.4 kHz when operating on binary patterns, while LED arrays can reach frequencies in the MHz range. It is noteworthy that an impressive SPI scheme utilizing an LED array has achieved a resolution of 32×32 pixels and a frame rate of 1000 fps [20]. Recently, a groundbreaking achievement in SPI imaging has been made using a DMD, enabling 2D and 3D imaging of periodic or reproducible scenes at an astounding frame rate of 2,000,000 fps [16]. However, it’s important to note that DMDs suffer from diffraction losses, while LED arrays exhibit significant divergence angles, both of which limit their applications in remote sensing. Additionally, an integrated optical phased array (OPA) chip, equipped with electro-optic phase-shifters, has been utilized as a modulation device to further enhance the refresh rate up to 100 MHz [22, 23]. Nonetheless, the OPA chip faces challenges such as limited emitting power and a complex manufacturing process, making its application in remote sensing a demanding proposition.

Furthermore, the choice of reconstructing algorithm plays a crucial role in the efficiency of SPI. For random speckle fields, early conventional algorithms primarily rely on intensity correlation techniques, such as the computational ghost imaging (CGI) [2], differential ghost imaging (DGI) algorithm [24] and the normalized ghost imaging (NGI) algorithm [25]. However, these intensity correlation algorithms often require oversampling to achieve clear images. As a result, compressive sensing (CS) algorithms have been developed and applied to single-pixel imaging, leading to the emergence of compressive sensing-based SPI (CS-SPI) [26]. CS-SPI, while capable of reconstructing images with sub-Nyquist sampling, can be computationally intensive and susceptible to noise due to the complexity of its calculations. Recently, SPI schemes based on deep learning have been introduced to improve imaging efficiency and accuracy [27–29]. Notably, in 2017, Lyu et al. proposed a physics-informed deep learning approach for ghost imaging known as GIDL [27]. GIDL utilizes a deep neural network (DNN) trained on traditionally CGI reconstructed images and ground truth data. Furthermore, in 2022, Wang et al. introduced a physics-enhanced deep learning approach for SPI [28]. This approach enables the incorporation of both data and physics priors into the inverse problem solvers used in SPI. In the same year, an untrained neural network (UNN) was employed to achieve far-field super-resolution SPI [29]. Notably, this method eliminates the need for pre-training on any specific data set and introduces a novel framework for the application of deep learning in SPI systems. By leveraging the capabilities of UNNs, this approach opens up new possibilities for improving the resolution and performance of SPI.

Building on the insights gained from OPA chip SPI schemes, we propose the utilization of a phase-controlled coherent fiber laser array as the illuminating source for SPI. The concept of employing a laser array for SPI illumination has been previously analyzed in theoretical studies [30–32]. However, to the best of our knowledge, practical implementation of collimated fiber laser arrays in SPI systems has not been reported. In our work, we arrange the fiber lasers in a compact hexagonal structure, which has found broad application in high-power coherent beam combination (CBC) [33, 34]. The hexagonal structure can yield high coherent combining efficiency in CBC due to the high fill factor with compact arrangement. In addition, the compact hexagonal structure can reduce the size of output laser array which makes it easier to process the following optic devices. The fiber laser array proposed in this study is controlled by LiNbO_3_ electro-optic phase modulators with a modulation bandwidth of 100 MHz. This configuration offers the potential to generate an illuminating light field with both high emitting power and a high-speed refreshing frequency. To further enhance the imaging quality and efficiency of this SPI system, an UNN based on SPI model is employed. The feasibility and robustness of the proposed SPI strategy are demonstrated in this paper through numerical simulations and experimental validations.

## Theory and numerical simulation

The compact hexagonal fiber laser array is broadly employed in CBC systems due to its ability to achieve a high fill factor, resulting in a high power ratio in the central lobe. Previous CBC schemes based on a compact hexagonal structure have successfully achieved 100 beams combining and 20 kW output powers [33, 34]. Therefore, a similar fiber laser array can be introduced in SPI to attain high emitting power and fast modulation capabilities for remote sensing applications. Unlike the CBC system, the SPI system primarily focuses on the spatial correlation properties of the illuminating light field, which can be evaluated using the normalized second-order intensity correlation function *g*^(2)^(*x*,* y*;* x*_0_,* y*_0_) [30].

1 $$g^{(2)} (x,y;x_{0} ,y_{0} ) = \frac{{\left\langle {I(x_{0} ,y_{0} )I(x,y)} \right\rangle }}{{\left\langle {I(x_{0} ,y_{0} )} \right\rangle \left\langle {I(x,y)} \right\rangle }},$$

where *I*(*x*_0_, *y*_0_) is the light intensity at the position (*x*_0_, *y*_0_) in cartesian coordinates. ⟨*I⟩ *means the ensemble average value of intensity *I* over time.

**Fig. 1:**
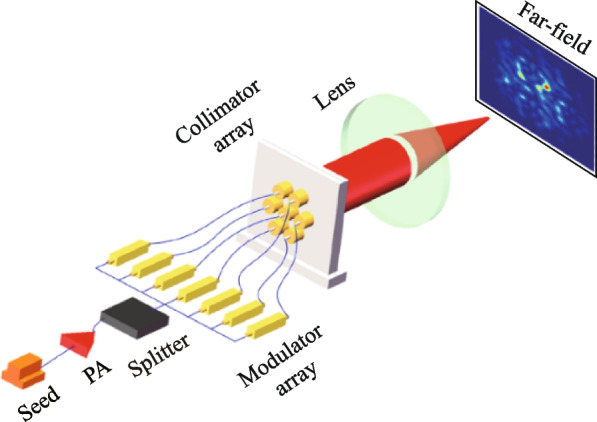
Schematic system of coherent fiber laser array

Figure 1 illustrates a typical hexagonal fiber laser array system. The system consists of a seed laser that undergoes pre-amplifier (PA) and is then split into multiple channels of fiber lasers. Each individual fiber laser is modulated by its own high-speed electro-optic phase modulator. The modulated fiber lasers are collimated using a collimator array and enter into free space. At the emitting plane, a focusing lens is positioned to combine the laser beams. Consequently, the light field at the focal plane of the lens can be considered as the far-field of the laser array, which also serves as the illuminating light field for the object in SPI.

The fiber laser array consists of several sub-arrays, as for the subarray, each laser beam can be regarded as Gaussian beam with size of *w*_0_. And each laser beam is truncated by a circular aperture with diameter of *D*. The light field at the emitting plane can be expressed by 

2 $$\begin{aligned}{E}_{e}\left(x,y,z=0\right) &=\sum\limits_{n=1}^{N}{A}_{n}\mathrm{ exp}\left(-\frac{{\left(x-{x}_{n}\right)}^{2}+{\left(y-{y}_{n}\right)}^{2}}{{w}_{0}^{2}}\right)\\ &\quad\times circ\left(\frac{\sqrt{{\left(x-{x}_{n}\right)}^{2}+{\left(y-{y}_{n}\right)}^{2}}}{\left(D/2\right)}\right){\text{exp}}\left({j\phi }_{n}\right)\!\end{aligned}$$

where *N* is the total number of sub-sources. *A*_*n*_ is the amplitude of the *n*th laser beam. (*x*_*n*_, *y*_*n*_) is the center coordinate of the *n*th laser beam. *ϕ*_*n*_ is the phase of the *n*th laser beam. *circ*(*r*_0_) denotes the function of the circular aperture. According to the Fraunhofer diffraction theory, the light field at the focal plane can be expressed as 

3 $$\begin{aligned} E_{r} (u,v,z = f) &= \frac{{{\text{e}}^{{{\text{i}}kf}} }}{{{\text{i}}\lambda f}}\int_{ - \infty }^{\infty } {\int_{ - \infty }^{\infty } {E_{e} (x,y,z = 0) }}\\ &\quad {{\cdot {\text{e}}^{{ - \frac{{{\text{i}}k}}{2f}(x^{2} + y^{2} )}} \cdot {\text{e}}^{{\frac{{{\text{i}}k}}{2f}[(u - x)^{2} + (v - y)^{2} ]}} } } {\text{d}}x{\text{d}}y. \end{aligned}$$

where *k* denotes the wavenumber of laser beam. *f* is the focal length of lens. *λ* is the wavelength of laser beam and (*u*, *v*) is the coordinate parameter at focal plane. Accordingly, the intensity distribution of light field at focal plane can be calculated as

4 $$I_{r} (u,v,z = f) = E_{r} (u,v,z = f) \cdot E_{r}^{ * } (u,v,z = f).$$

**Fig. 2:**
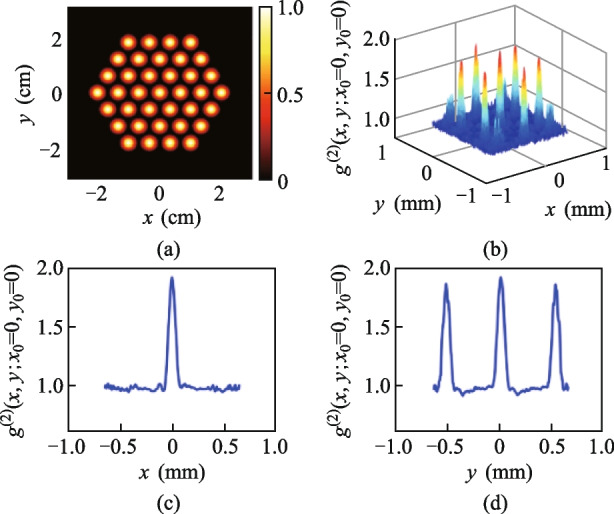
**a** 37 sub-channels hexagon fiber laser array. **b** 2D distribution of *g*^(2)^(*x*,* y*;* x*_0_*=* 0,* y*_0_* =* 0) on the focal plane. **c** and **d** Cross-section of *g*^(2)^(*x*,* y*;* x*_0_* =* 0*, y*_0_* =* 0) along the (*x, y =* 0) direction and the (*y, x =* 0) direction

Based on the aforementioned analysis, the normalized second-order intensity correlation function *g*^(2)^(*x*,* y*;* x*_0_,* y*_0_) at the focal plane can be calculated and the results are shown in Fig. 2. In this study, we consider a laser wavelength of 1064 nm and a focal length of 3 m for illustrative purposes. The hexagonal array configuration consists of a total of *N* = 37 laser beams, as depicted in Fig. 2a. The size of single Gaussian laser beam *w*_0_ and the diameter of circular aperture *D* are set to be *w*_0_ = *D*/2 = 3 mm. The center-to-center distance *L* between two adjacent laser beams is determined as 7 mm. The fill factor of the hexagon array can be calculated to be *D/L* = 0.86. To obtain the accurate *g*^(2)^(*x*,* y*;* x*_0_,* y*_0_) distribution, the sampling number of light field is set to be 3000. Figure 2b displays the 2D distribution of *g*^(2)^(*x*,* y*;* x*_0_*= *0,* y*_0_= 0) on the focal plane. Figure 2c and d present the cross-sections along the (*x*, *y =* 0) and the (*y*, *x =* 0) directions, respectively. It shows that the distribution of *g*^(2)^(*x*,* y*;* x*_0_* = *0,* y*_0_* =* 0) exhibits spatial periodicity, and the sidelobes of *g*^(2)^(*x*,* y*;* x*_0_*=* 0,* y*_0_* =* 0) are equal to the main peak in the position (*x =* 0*, y =* 0).

**Fig. 3:**
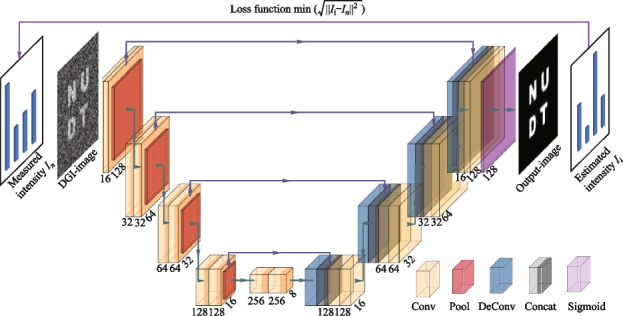
Network optimization process of UNN-SPI

It has been proven that the spatial periodicity of illuminating light fields can negatively impact the imaging quality of SPI in reference [30]. To address this issue, we propose the integration of an untrained neural network (UNN) with the SPI physical model to optimize the quality of reconstructed images [29]. Remarkably, this neural network exhibits superior performance, particularly at low sampling ratios, and does not require pre-training on any specific data set. The imaging process based on the UNN algorithm is depicted in Fig. 3 and can be summarized in four steps. First, a rough image is reconstructed by the DGI algorithm through combining the illuminating fields and the measured intensity *I*_*n*_. Secondly, the DGI image serves as input to the randomly initialized UNN, generating a high-quality output image. Thirdly, the output image is treated as the object and used to calculate the estimated intensity *I*_*i*_ through the SPI technique. Finally, we calculate the root-mean-square error (RMSE) between *I*_*i*_ and *I*_*n*_, and this serves as loss function guiding the network optimization until reaching the minimal value. This iterative process leads to the improvement of the output image quality and a better reconstruction outcome.

**Fig. 4:**
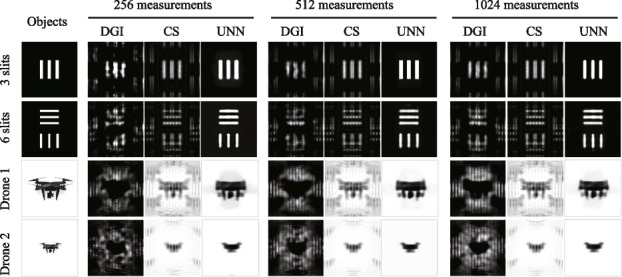
Simulated SPI results based on hexagon fiber laser array

Using the algorithms mentioned above, we conducted simulations of SPI results utilizing a hexagonal fiber laser array, as depicted in Fig. 4. Both binary and grayscale images with resolution of 128 × 128 pixels are selected as the objects, including binary 3 slits, binary 6 slits and two gray drone images of different sizes (Drone 1 and Drone 2). The size of the 3 slits is half that of the 6 slits, and similarly, the size of Drone 2 is half that of Drone 1. The reconstruction algorithms utilized in this study include the differential ghost imaging (DGI) algorithm [24], the sparse representation prior compressive sensing (CS) algorithm [30], and the untrained neural network (UNN) algorithm [29]. The sampling measurements are conducted with settings of 256, 512, and 1024. As observed in the results, increasing the sampling measurements leads to clearer reconstructed images with enhanced details and reduced noise. Additionally, it is evident that the images reconstructed using the DGI and CS algorithms exhibit noticeable periodicity. By contrast, the UNN-SPI method offers the ability to reconstruct images without the presence of periodicity, resulting in images with superior levels of detail. Table 1 presents the root-mean-square error (RMSE) values of the reconstructed SPI results, which demonstrates decreasing RMSEs as the sampling measurement increases. And the RMSEs of UNN-SPI are obviously lower than those of DGI-SPI and CS-SPI. Notably, the reconstructed images of UNN-SPI exhibit excellent quality even with 256 sampling measurements, corresponding to a sampling ratio of 1.6%. This finding highlights the effectiveness of the UNN algorithm in enhancing both the efficiency and image quality in SPI applications based on fiber laser arrays.

**Table 1:** RMSE values of the above SPI results

Object	256 measurements			512 measurements			1024 measurements		
	DGI	CS	UNN	DGI	CS	UNN	DGI	CS	UNN
3 slits	0.1710	0.1427	**0.0551**	0.1594	0.1401	**0.0544**	0.1489	0.1378	**0.0528**
6 slits	0.2239	0.1958	**0.1328**	0.2092	0.1921	**0.1158**	0.2064	0.1877	**0.1112**
Drone 1	0.8270	0.2023	**0.1343**	0.8067	0.1989	**0.1289**	0.7878	0.1950	**0.1260**
Drone 2	0.8543	0.0805	**0.0627**	0.8139	0.0785	**0.0622**	0.7802	0.0769	**0.0597**

**Fig. 5:**
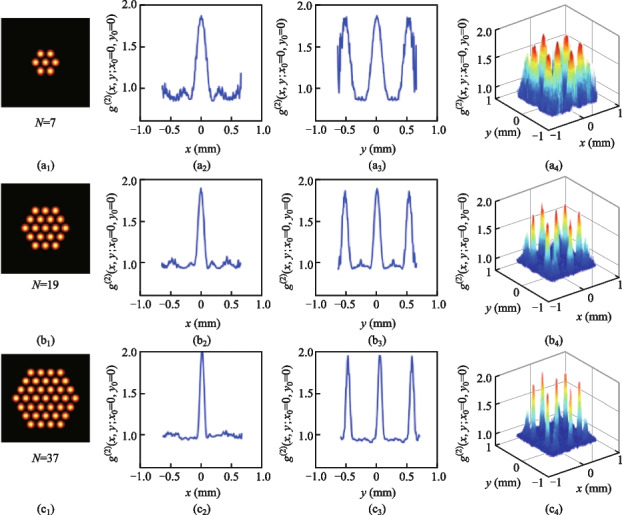
Variation of *g*^(2)^(*x*,* y*;* x*_0_* =* 0*, y*_0_* =* 0) with coordinates (*x* and *y*) when the number of laser array sub-channels is 7, 19, or 37. **a**_1_–**c**_1_ The number of laser array sub-channels are 7, 19, 37. **a**_2_–**c**_2_ Cross-section of *g*^(2)^(*x*,* y*;* x*_0_* =* 0,* y*_0_* =* 0) along the (*x, y =* 0) direction. **a**_3_–**c**_3_ Cross-section of *g*^(2)^(*x*,* y*;* x*_0_* =* 0,* y*_0_* =* 0) along the (*y, x =* 0) direction. **a**_4_–**c**_4_ 2D distribution of *g*^(2)^(*x*,* y*;* x*_0_*=* 0,* y*_0_* =* 0) on the focal plane

**Fig. 6:**
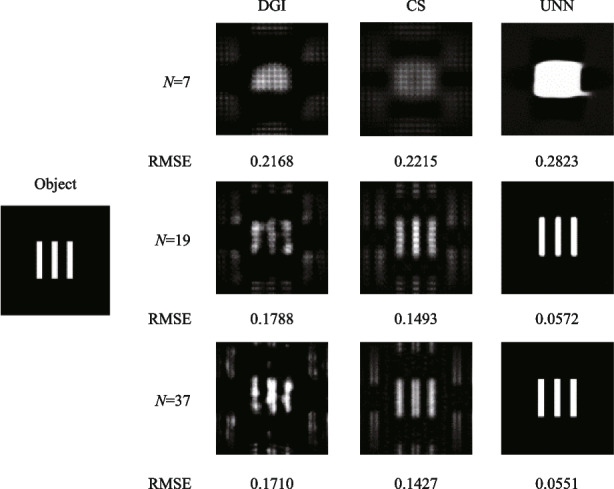
Simulated SPI results when the number of laser array sub-channels is 7, 19, or 37, respectively

The above analysis has proved that the hexagon laser array can achieve excellent SPI results by using the UNN reconstruction algorithm. Next, we will discuss how to design the hexagon laser array to obtain better SPI quality. The main influence factors are the sub-channel’s number and the compactness of the hexagonal laser array. Figure 5 shows the normalized second-order intensity correlation function *g*^(2)^(*x*,* y*;* x*_0_,* y*_0_) when the number of laser array sub-channels *N* is set to values of 7, 19 and 37. The fill factor of the hexagon array is kept to be *D*/*L* = 0.86. Figure 6 shows the corresponding SPI results of a 3 slits image with a resolution of 128 × 128 pixels and sampling ratio of about 1.6%. In Fig. 5, when the number *N* increases, then the linewidth of *g*^(2)^(*x*,* y*;* x*_0_,* y*_0_) decreases. The *g*^(2)^(*x*,* y*;* x*_0_,* y*_0_) can be regarded as the point spread function of SPI system. The narrower linewidth can lead to a higher resolution of SPI reconstructing images. Therefore, the laser array with more laser beams can achieve better SPI results in Fig. 6.

**Fig. 7:**
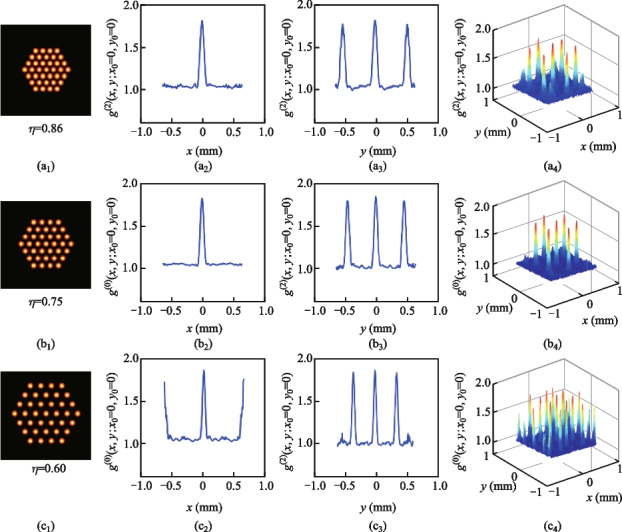
*g*^(2)^(*x*,* y*;* x*_0_* =* 0, *y*_0_*=* 0) when the fill factors of the laser array are 0.86, 0.75, or 0.60

**Fig. 8:**
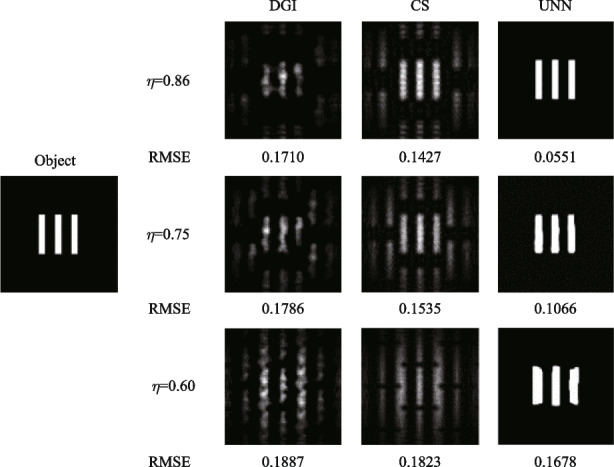
Simulated SPI results when the fill factor of laser array is 0.86, 0.75, or 0.60

In addition, the compactness is another important factor influencing the SPI results. The compactness of the hexagon laser array mainly depends on the fill factor values. Here we set the center-to-center distance *L* between two adjacent laser beams to be 7, 8, and 10 mm. The corresponding fill factors can be calculated to be 0.86, 0.75, and 0.60, respectively. The sub-channel’s number of laser array *N* is set to be 37 for convenience. The *g*^(2)^(*x*,* y*;* x*_0_,* y*_0_) distributions of different laser arrays are shown in Fig. 7. The corresponding simulated SPI results are shown in Fig. 8. When the fill factor increases from 0.60 to 0.86 then the laser array becomes more compact, and the period of *g*^(2)^(*x*,* y; x*_0_*, y*_0_) also increases. Then the number of peaks decreases in the illuminating field. Therefore, the more compact laser array can have better SPI results, as can be observed in Fig. 8. In addition, the more compact laser array can offer significant convenience for the optical system due to the smaller size of output lasers.

## Experimental results

**Fig. 9:**
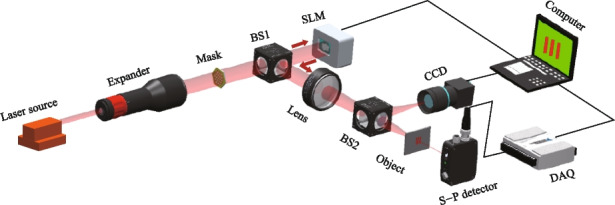
Experimental set up based on SLM system

To validate the feasibility of the hexagon laser array and UNN-SPI algorithm, a concept demonstration experiment system is constructed utilizing a spatial light modulator (SLM). The experimental setup is depicted in Fig. 9. In the setup, a single-frequency laser source operating at a wavelength of 1064 nm and with a diameter of 0.2 cm is first expanded by a beam expander, resulting in a 10-fold increase in the diameter of the beam. Next, the expanded laser beam traverses a mask comprising 37 sub-apertures arranged in a hexagonal array configuration. The design of the hexagonal array on the mask ensures a fill factor of 0.86, effectively transforming the Gaussian laser into a hexagonal laser array. Subsequently, the laser array passes through a beam splitter (BS1) and undergoes random modulation using a phase-type spatial light modulator (SLM). The SLM allows for phase modulation within a range of 0 to 2π, enabling precise control over the phase distribution of the laser array. Then the modulated laser array is reflected by BS1 and is focused using a lens with a focusing length of 1 m. The focused light is further divided by another beam splitter (BS2). A CCD camera positioned on the focal plane detects and records the distribution of the reflected light, serving as a means to capture the light field distribution. Simultaneously, the transmitted light illuminates an object that is also situated on the focal plane. The transmitted light from the object is collected by a single-pixel (S-P) detector. A computer system is employed to control the phase modulation of the SLM and facilitate synchronized data acquisition (DAQ) from both the CCD camera and the S-P detector.

**Fig. 10:**
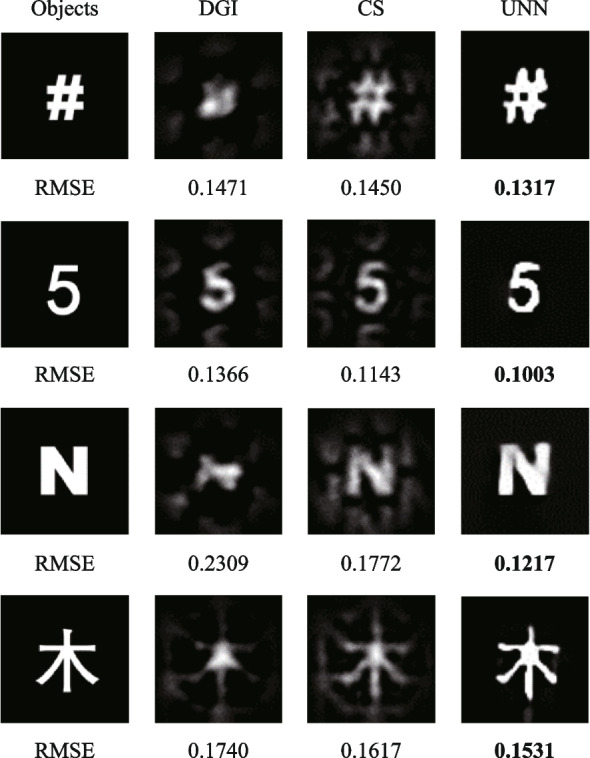
Experimental SPI results based on SLM system

A self-developed software is used to control the SPI process based on the computer. First, the computer controls the SLM to provide refreshing random phase to the laser array. During each phase modulation, the CCD camera and the S-P detector commence data collection simultaneously. The CCD camera records the intensity distribution of the illuminating light fields and transfers the data to the computer directly. The S-P detector records the light intensity from object and saves the acquired data to the computer by DAQ. By combining the light fields captured by the CCD camera with the light intensities measured by the S-P detector, we reconstruct the object image using different algorithms at a sampling ratio of 6.25%. The SPI results are depicted in Fig. 10, showing the successful reconstruction of various objects, including a computer symbol, a mathematical number, a Latin character, and a Chinese character. The reconstructed images possess resolution of 64 × 64 pixels. Notably, even with a sampling ratio of 6.25%, the UNN-SPI technique demonstrates its ability to accurately reconstruct all the objects, as clearly observed in the experimental results. To provide a comprehensive comparison, the results of DGI and CS-SPI algorithms are also calculated. However, it is evident that the reconstructed images obtained from DGI and CS-SPI suffer from significant spatial periodicity issues, which severely compromise the overall imaging quality. The UNN-SPI results hold the lowest RMSE values for all the objects. This observation highlights the clear advantage of the UNN-SPI technique in producing superior reconstruction results without the presence of such periodicity-related impairments.

**Fig. 11:**
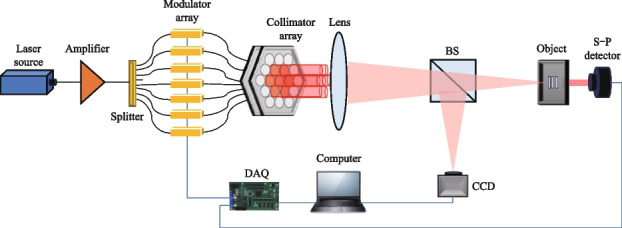
Experimental set up of fiber laser array system

Furthermore, a SPI system based on a fiber laser array is established, as depicted in Fig. 11. Initially, a single-frequency linearly-polarized fiber laser with a central wavelength of 1064 nm is amplified to a power of 1 W using a fiber amplifier. The amplified laser is then divided into 8 sub-channels through a 1 × 8 splitter. 7 of these sub-channels are subjected to modulation using a LiNbO_3_ electro-optic phase modulator array and are subsequently connected directly to a homemade collimator array. The collimator array consists of two main components: a fiber connector array and a collimating lens array. The fiber connector array features a flexible hinge structure, allowing for three-dimensional adjustments of the fiber tip’s position to ensure it aligns precisely with the focal point of the collimating lens [33]. Subsequently, the fiber laser array produces collimated output beams. The collimating lens employed in the system has a diameter of 23 mm, with adjacent lenses separated by a distance of 25 mm. Through calculations, the fill factor of the laser array is determined to be 92%. After passing through the collimating lens, the beam waist diameter is measured to be 21 mm. Following the collimator array, a focusing lens is utilized to combine the individual laser beams at the focal plane. Just before reaching the focal plane, the laser beam is split into two components using a beam splitter (BS). The reflected portion of the laser beam is captured by a CCD camera, which records the distribution of the light field. Simultaneously, the transmitted laser beam illuminates the object, and a single-pixel detector is employed to measure the light intensity after interaction with the object. To facilitate control and data acquisition, a data acquisition card (DAQ) is utilized to control the phase modulator array and obtain data from the single-pixel detector. The data acquisition processes of both the DAQ and the CCD camera are coordinated and controlled by a computer, ensuring synchronized operation and efficient data collection.

**Fig. 12:**
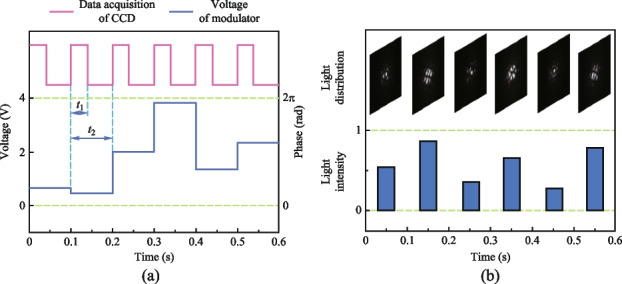
Applied voltage on phase modulator and the data acquisition of CCD camera. **a** The process of data acquisition and phase modulation. **b** The recorded light field distributions captured by the CCD camera corresponding to the light intensities measured by the S-P detector

**Fig. 13:**
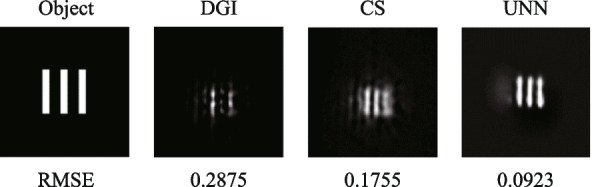
Experimental SPI results based on fiber laser array

The execution frequency of the data acquisition card (DAQ) for controlling the phase modulator is capable of reaching a maximum of 2 kHz. However, in this case, it is set to operate at 10 Hz due to the limited refresh rate of the CCD camera. Figure 12a illustrates the process of data acquisition and phase modulation. The LiNbO_3_ electro-optic phase modulator, with a half-wave voltage of 2 V, is controlled by the DAQ, which generates random voltage signals ranging from 0 to 4 V. This voltage range allows for precise phase modulation within the range of 0 to 2π, enabling accurate control of the laser beam’s phase characteristics. During each modulation cycle, which lasts for a duration of 0.1 s (*t*_2_), both the CCD camera and the single-pixel detector collect data simultaneously. The CCD camera’s exposure time is set to 0.04 s (*t*_1_) for each data acquisition, ensuring optimal image capture. Figure 12b illustrates the recorded light field distributions captured by the CCD camera, as well as the corresponding light intensities measured by the single-pixel detector. The entire process of data acquisition and phase modulation is managed by a self-developed software running on the computer. In this study, we have chosen a set of three slits as the representative object. By sampling 256 illuminating light fields and capturing the corresponding interacting light intensities, we proceed to reconstruct the object using various algorithms. The SPI results are presented in Fig. 13. The reconstructed images obtained have a resolution of 128 × 128 pixels, with a corresponding sampling ratio of 1.6%. Notably, the UNN-SPI technique exhibits superior image quality in comparison to both DGI and CS-SPI methods. The reconstructed image produced by UNN-SPI shows a distinct profile and minimal background noise, highlighting the feasibility and efficiency of utilizing the UNN-SPI approach based on a fiber laser array. These results underscore the potential and effectiveness of this technique for high-quality image reconstruction applications.

## Conclusions

In conclusion, we have successfully employed a compact fiber laser array with a hexagonal structure as the illuminating light source in SPI experiments. Given the spatial periodicity present in the generated illumination light field, we introduce the UNN-SPI technique for object reconstruction. Theoretical simulations confirm the feasibility of both the proposed fiber laser array and the UNN-SPI algorithm. Additionally, we establish an SLM system and a phase-controlled fiber laser array system for further experimental validation. The experimental results demonstrate that the UNN-SPI technique outperforms traditional methods such as DGI and CS-SPI, enabling clear image reconstruction even at remarkably low sampling ratios. Future efforts will be directed toward enhancing the sampling speed of the CCD camera to achieve faster or real-time SPI. Moreover, considering the broad application of phase-controlled fiber laser arrays in high-power coherent beam combining, we believe that our proposed approach provides a novel and powerful illuminating source for SPI, with potential applications in fields such as remote sensing. Next, we will concentrate on researching the influence of atmospheric turbulence to improve the imaging distance of SPI in the actual environment.

## Data Availability

The data that support the findings of this study are available from the corresponding author, upon reasonable request.
